# Therapeutic Use of Botulinum Neurotoxins in Dermatology: Systematic Review

**DOI:** 10.3390/toxins13020120

**Published:** 2021-02-05

**Authors:** Emanuela Martina, Federico Diotallevi, Giulia Radi, Anna Campanati, Annamaria Offidani

**Affiliations:** Dermatological Clinic, Department of Clinical and Molecular Sciences, Polytechnic Marche University, 60020 Ancona, Italy; ema.martina@gmail.com (E.M.); federico.diotallevi@pm.univpm.it (F.D.); radigiu1@gmail.com (G.R.); annamaria.offidani@ospedaliriuniti.marche.it (A.O.)

**Keywords:** botulinum toxin, dermatology, skin diseases, focal idiopathic hyperhidrosis, Hailey–Hailey disease, epidermolysis bullosa simplex Weber–Cockayne type, Darier’s disease, pachyonychia congenita, suppurative hidradenitis, aquagenic keratoderma, alopecia, psoriasis, notalgia paresthetica, facial erythema and flushing, oily skin, Raynaud phenomenon

## Abstract

Botulinum toxin is a superfamily of neurotoxins produced by the bacterium Clostridium Botulinum with well-established efficacy and safety profile in focal idiopathic hyperhidrosis. Recently, botulinum toxins have also been used in many other skin diseases, in off label regimen. The objective of this manuscript is to review and analyze the main therapeutic applications of botulinum toxins in skin diseases. A systematic review of the published data was conducted, following Preferred Reporting Items for Systematic Reviews and Meta-Analysis (PRISMA) guidelines. Botulinum toxins present several label and off-label indications of interest for dermatologists. The best-reported evidence concerns focal idiopathic hyperhidrosis, Raynaud phenomenon, suppurative hidradenitis, Hailey–Hailey disease, epidermolysis bullosa simplex Weber–Cockayne type, Darier’s disease, pachyonychia congenita, aquagenic keratoderma, alopecia, psoriasis, notalgia paresthetica, facial erythema and flushing, and oily skin. Further clinical trials are still needed to better understand the real efficacy and safety of these applications and to standardize injection and doses protocols for off label applications.

## 1. Introduction

Botulinum toxin (BoNT) is a superfamily of neurotoxins able to block the release of acetylcholine and many other neurotransmitters from presynaptic vesicles by cleavage of target proteins of the SNARE.

BoNT-A has a long history of therapeutic application in idiopathic focal hyperhidrosis, and aesthetic medicine with a strong efficacy and safety profile [1,2,3,4,5,6,7,8]. The great potential therapeutic application of BoNT-A has driven clinicians to evaluate its therapeutic potentiality in many other cutaneous diseases. The aim of this review is to collect and analyze the published data concerning the most relevant label and off-label indications of botulinum toxin in dermatology.

## 2. Results

**Figure 1:**
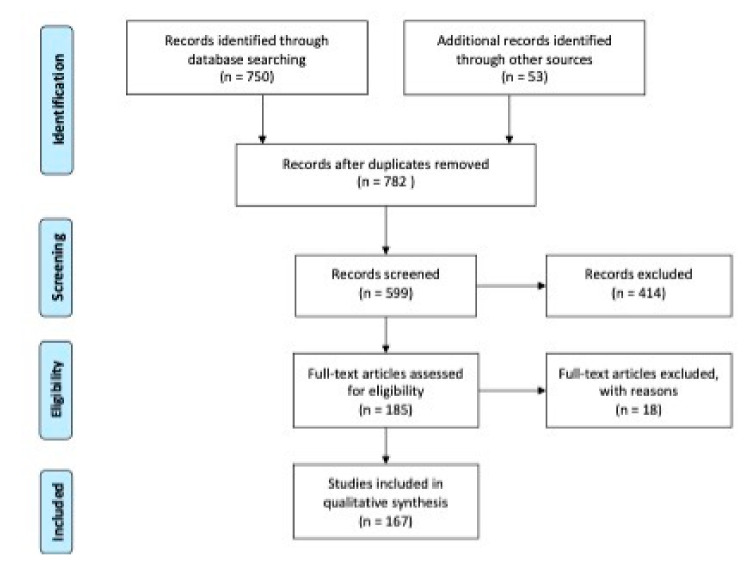
Preferred Reporting Items for Systematic Reviews and Meta-Analysis (PRISMA) on botulinum toxin (BoNT)-A in dermatology. Research dates range from 1994 to 2020. Reprinted with permission of reference [9]. Copyright 2009 Moher et al. Website: https://www.ncbi.nlm.nih.gov/pmc/articles/PMC2707599/figure/pmed-1000097-g001/ (accessed on 7 November 2020).

The PRISMA study flowchart is shown in Figure 1 [9]. Our search identified 782 records after removing duplicates. After scanning the titles and abstracts, 414 citations were excluded. After examining the full text, 185 case-control, case series studies, randomized controlled trials were considered eligible, and among them 167 were included in this study.

Diseases for which BoNTs could have therapeutic potentials could be summarized as follows:

Sweat glands disorders

-   Idiopathic hyperhidrosis
-   Chromhidrosis
-   Bromhidrosis

Facial erythema and flushing

Raynaud phenomenon

Pompholyx

Eccrine nevus

Postherpetic neuralgia

Oily skin

Notalgia paresthetica

Hailey–Hailey Disease

Genodermatoses

-   Epidermolysis bullosa simplex, Weber–Cockayne type
-   Darier disease
-   Pachyonychia congenita

Hidradenitis suppurativa

Aquagenic keratoderma

Alopecia

-   Alopecia areata
-   Androgenetic alopecia

Psoriasis

### 2.1. Sweat Gland Disorders

#### 2.1.1. Idiopathic Hyperhidrosis

Idiopathic hyperhidrosis (IH) is a chronic autonomic disorder characterized by an overproduction of sweat [10]. It is easy to comprehend how IH affects the quality of life, leading to emotional and social shame, as well as professional, physical and psychological impairment. Focal and multifocal IH is also a pediatric disease, associated with severe negative quality of life [11].

At present, little is known about the causes of IH. A family history is commonly reported by patients, which suggests the role of genetic transmission [12]. A familial variant with autosomal dominant transmission is now identified in some families that express an anomaly in chromosome 14q [13].

IH usually starts in childhood and affects 0.6%–1% of the population, its prevalence may differ according to the definition of hyperhidrosis [14].

Excessive sweating lasting at least six months without any clear cause and possessing at least two of the following characteristics are the diagnostic criteria for IH: Impaired everyday activities, bilateral and reasonably symmetrical sweating pattern occurring at least once a week, age of onset younger than 25 years, cessation of focal sweating during sleep, or positive family history [10].

The diagnosis of IH can only be considered after secondary causes of excessive sweating have been excluded such as drugs, toxins, and systemic diseases (endocrine, neurologic or metabolic conditions, malignancies) [14]. In 90% of IH cases, commonly affected areas include the axillae, palms, soles, or craniofacial regions [15].

There are various treatment options currently available to manage IH [16]. Initial treatment consists in lifestyle and behavioral recommendations such as avoiding emotional stress, spicy foods, and alcohol. The use of wide clothing, natural fabrics, and leather footwear may also help. Antiperspirants are considered the first-line therapy for IH. Specifically, aluminum chloride hexahydrate is the most common and effective topical medications used for mild to moderate IH [17]. However, the use of these topicals can have some disadvantages such as irritant or allergic contact dermatitis, inadequate long-term efficacy.

In asymptomatic volunteers, the sweat reducing effect of botulinum neurotoxin (BoNT) was first observed [18]. By deactivating SNARE proteins, BoNT prevents the release of acetylcholine and a variety of other neurotransmitters from presynaptic vesicles [10]. Neural activity of eccrine sweat secretion is regulated by acetylcholine, and therefore, BoNT injections decrease sweat secretion [19]. Four types of BoNTs are available for clinical use: OnabotulinumtoxinA (A/Ona, Botox), incobotulinumtoxinA (A/Inco, Xeomin), abobotulinumtoxinA (A/Abo, Dysport), and rimabotulinumtoxinB (B/Rima, Myobloc). These toxins use different presynaptic proteins for their site of action [20]. Intradermal injections for hyperhidrosis treatment are usually carried out in a grid pattern with a small needle (gauge 30) to the depth of few millimeters, with 2–2.5 units of toxin administered at each site. To reduce the pain, the injection is done after applying a local anesthetic spray or ice spray [18]. The most common complaint is pain caused by injections [21], especially in palms and plantar areas. Some strategies can avoid this problem, including needle-free anesthesia [22], cryoanalgesia, vibration analgesia, pocketed microneedles [6], topical anesthetics, dilution with lidocaine, sedation, intravenous regional anesthesia, and nerve blocks [16]. Skiveren et al. investigated the influence of needle size on the pain associated with BoNT injections, comparing 27 G and 30 G needle. The Authors’ findings indicate that needle perforation may not necessarily be the most important contributor of the pain associated with injections. The study advanced the hypothesis that other factors, such as hydrostatic pressure on the surrounding tissue and activation of nociceptors by chemicals in the solution, can provoke pain [21]. Research conducted in 2013 by our working group has suggested that the injection procedure for idiopathic palmar hyperhidrosis with a patent medical device can reduce the pain and is preferred by patients compared to wrist block [6].

As is well known, BoNT injections are very effective in the treatment of axillae with a high safety profile and a well-tolerated procedure [23]. A number of trials found evidence that the injection of 50 units (U) of BoNT-A per axilla is effective in IH [18,19,20,21,22]. Marcella et al. observed that 35U per axilla can be effective and appreciated in female patients with moderate hyperhidrosis, suggesting the possibility of a tailored treatment [24]. Based on these results, an established level of evidence (Level A) exists only for BoNT-A treatment of idiopatic axillary hyperhidrosis [10].

Concerning the duration of symptoms relief, the effects of BoNT-A last for 4–9 months on average in axillary use [25]. D’Epiro et al. reported a mean of 7.2 months symptoms-free period [26]. As is already known in clinical practice, a common finding is the increase in the length of efficacy of botulinum toxin A injections with repeated injections. Lecouflet et al. published a study on 83 subjects with idiopathic axillary hyperhidrosis and reported a statistically significant increase in the duration of efficacy with the repetition of injections [27]. The Authors observed similar results in 23 patients suffering from idiopathic palmar hyperhidrosis [28]. They speculated that the increase in duration and efficacy of injection repetitions were related to the gradual regeneration of the axon terminal of the motor neuron. Berthin and Maillard recently confirmed these findings with a retrospective 15-years study on 117 patients [29].

Curettage and tumescent liposuction have been proposed as an alternative to BoNT-A in the treatment of axillary IH [30]. At the junction between the dermis and hypodermis, where most sweat glands are placed, a cannula is inserted. A side-by-side study was conducted on 20 subjects in 2013 by Ibrahim et al. [31]. Neurotoxin injections were statistically significantly superior to suction-curettage for reduction of idiopathic axillary hyperhidrosis in a 6-month follow up period. Moreover, patients declared a marked preference for the botulinum toxin therapy.

Idiopathic palmar hyperhidrosis is a disabling condition of unknown etiology, although hyperactivity of the sympathetic fibers that pass through the thoracic sympathetic ganglia T2 and T3 has been reported [32]. Topical medications [17], iontophoresis [33], oral oxybutynin [34], and surgical management [16] have been proposed for the treatment of this focal hyperhidrosis.

Oxybutynin, an anticholinergic oral medication, has been employed in a number of cases for the treatment of hyperhidrosis, especially multifocal or generalized [34]. Oxybutynin chloride is actually indicated in persons with an uninhibited neurogenic or reflex neurogenic bladder for the relief of signs of bladder instability associated with voiding. However, an increasing amount of literature supports the therapeutic use of oxybutynin in primary hyperhidrosis at a dose of 5–15 mg daily [35,36,37,38]. Relatively mild side effects include dry mouth, headache, constipation and urinary retention, particularly when the daily dose of oxybutynin reaches 15 mg [36]. Current research seems to indicate that the safety and efficacy profile of oxybutynin in hyperhidrosis allows the association with BoNT-A treatment. Specifically, we have recently examined the efficacy of sequential administration of oral oxybutynin chloride after BoNT-A injections vs. oral oxybutynin chloride in monotherapy in patients with primary palmar hyperhidrosis [39]. Moreover, we evaluated if the sequencing approach could allow the control of hyperhidrosis with lower dose of oral oxybutynin. Our findings showed that the combination therapy helps patients to extend disease-free survival from conventionally observed of 2 to 8 months after injection of BoNT-A to 52 months with a high safety profile and fewer side effects.

Surgical approach (sympathicotomy and thoracic sympathectomy) has been suggested in severe hyperhidrosis after failure of other strategies, but compensatory hyperhidrosis remains a dramatic adverse event [40].

Concerning the use of BoNT-B in the treatment of idiopathic palmar hyperhidrosis, there is a considerable debate on the conversion and diffusion of type B toxin compared to BoNT-A [41,42]. Basciani et al. proposed a total dose of 5000 IU of BoNT-B (rimabotulinum toxin b) per palm diluted with 10 mL of 0.9% iodine solution [43]. In clinical practice, a conversion ratio of 1:50–100 (BoNT-A vs. BoNT-B, for the commercially product Botox and Neurobloc, respectively) is generally considered in treating autonomic disorder [41]. Despite the anhidrotic effect of this injection regimen, the treatment IH with BoNT-B has been evaluated in a limited number of studies, compared with BoNT-A. The significant difference is probably due to the higher risk of immunogenicity correlated to the higher protein load and total cumulative dose injected [44]. Many factors influence the immunogenicity of BoNTs, related to the product itself (manufacturing processes, toxin source, inactive toxin, antigenic protein load, accessory proteins and excipients), to treatment (dose, treatment intervals, previous exposure, or vaccination) and to the host (genetic predisposition) [44]. Since primary hyperhidrosis is a chronic condition, it’s extremely important to reassure the patient about the maintenance of efficacy in subsequent treatments. High doses of BoNT-A injected intradermally in one session increase also the risk of systemic neurological side effects. Kouris et al. reported the case of a young woman treated with 400 BoNT-A totally in one session for palmoplantar hyperhidrosis who experienced three days after the injection dizziness, headache, nausea, muscular weakness, muscular pain, inability to maintain support, difficulty in opening her eyelids, weakness of eye focus, and somnolence [45]. The symptoms presented progressive improvement and receded completely after six weeks. Generalized symptoms can be explained with the local uptake and retrograde axonal transport via the spinal motor neurons or a systemic distribution via the blood circulation. Other factors can be related to systemic side effects: Frequency of injection visits, wrong technical procedure, idiosyncratic reactions, genetic predisposition, low weight, gender and anatomical differences of the palms between sexes. The conventional regimen with BoNT-A requires 100U reconstituted with 5 mL of 0.9% sterile saline injected intradermally in each palm in approximately 25 sites No specific gender accommodation has to be done. The procedure can be more comfortable with the use of small needles (30 G), cryoanalgesia or with the wrist block, as we discussed before. The reported duration of efficacy differs between studies, from 2 to 22 months, related also to the dose injected [1,46]. A common side effect associated with palmar BoNT-A injections is temporary weakness of the thenar eminence muscles, demonstrated by grip weakness. An interesting research on this topic has been conducted on a large population (474 patients); the results showed that the muscle weakness was the second most frequent side effect within the first week after injection and that women experienced the discomfort twice more frequently than men. The interpretation provided by the authors concerns anatomical differences, works or recreational habits that differ by gender [47]. These data are of particular interesting in clinical practice and suggest a gender-related dosage and injection pattern. The treatment of plantar hyperhidrosis is less effective than in other sites, with approximately 50% of patients dissatisfied. The procedure can be more painful and the sweat reduction becomes significant after approximately 2 weeks [48].

#### 2.1.2. Chromhidrosis

An unusual disease characterized by the secretion of pigmented sweat is chromhidrosis. The color can be yellow, green, blue, or black; the face or armpits are most commonly afflicted by the disease. The condition is extremely disabling and causes shame in patients [49]. Little is known about of the etiopathogenesis of the disease and if the altered function is due to the apocrine or to the eccrine glands is still debated. After their first case successfully treated with BoNT-A, Wu et al. hypothesized an apocrine-related pathogenesis, although apocrine glands are historically known not to respond to cholinergic stimulation [50]. In contrast, because of the strong reaction to BoNT-A therapy, Matarasso thought chromhidrosis an eccrine-related disorder [51]. While axillary chromhidrosis seems to be completely controlled with BoNT-A injections [50], the cheek site appears to be more resistant or partially responsive [52]. An indication of this discrepancy can be seen in the greater dilution of the toxin, in order to produce the optimal effects without the possibility of injections near the eye region; the procedure in the cheek area requires further standardization in terms of dilution and doses.

#### 2.1.3. Bromhidrosis

The term bromhidrosis means a disease in which, owing to the interactions between apocrine gland secretions and bacteria, body odor is unpleasant. Sixty-seven patients with axillary bromhidrosis were enrolled in 2012 by He et al. 50 U of BoNT-A was administered into each axilla, and follow up was performed every month. The investigators recorded that 73.1% (49/67) of patients had malodor eliminated and that BoNT-A therapy was successful in patients with a strict association between sweating and malodor. Therefore, the key reason for BoNT-A therapy is near positive association between malodor and sweating [53].

Wu et al. recently published the results of a prospective randomized clinical study in association with histological analysis of sweat glands in axillary bromhidrosis treated with BoNT-A [54]. The mean degree of malodor and mean amount of sweat in the BoNT-A-treated axilla was substantially lower at 3 months after treatment than in the control axilla. Apocrine sweat glands with atrophic changes and hypoplasia in the treated axilla were observed in histological samples. He et al. enrolled 53 patients affected by secondary axillary bromhidrosis following various surgical modalities; they were treated with 50U of BoNT-A diluted in 2 mL of saline and reached a median disease-free survival of 6 months [53]. In adolescent age, bromhidrosis heavily impairs quality of life and relationship. Wang et al. recruited 62 adolescent patients with primary axillary bromhidrosis and 50 U of BoNT-A was administered in each axilla. The treatment was effective with a good satisfaction (51/62 patients ranked the BoNT-A treatment to be very good or good) but the efficacy lasts less than 4 weeks in 38.7% of subjects [55]. In both males and females, foul genital odor is another distressing problem, with a great effect on the quality of life. Bacterial infection of the genital skin or vaginal mucosa is the key cause of genital odor, although in certain cases, the contact between local sweat and some species of bacteria is the explanation. Lee et al. identified a rare case of a female patient with a history of many years of bad genital odor unsuccessfully treated with antibacterial soaps, perfume and antimicrobial agents [56]. Genital infections were excluded. The patient was treated with BoNT-A injections in 40 different sites (2.5 mU/0.1 mL per site) of the genital hair-bearing area. She experienced a significant decrease in the odor for 9 months. Table 1 resumes all the cases reviewed.

### 2.2. Facial Erythema and Flushing

The possible therapeutic action of botulinum toxin type A for facial erythema and redness has been discussed in several recent reports including both some interesting case reports [57,58,59] and equally discouraging results [60,61,62]. In 2011 Odo et al. [63] conducted a study with the aim to evaluate the reduction of the discomfort of menopausal hot flashes by the intradermal injection of abo- botulinum toxin A. They enrolled 60 female patients with menopausal hot flashes and they treated the affected area (scalp, face, neck, and chest) with a total of 500 U of abo- botulinum toxin A, diluted in 3.2 mL of saline solution; 6.2-U were injected into each selected skin site. The control group was treated with saline at the same volume of 0.04 mL per injection point. The Minor test was used to detect areas with exceeding sweating and patients noted in a diary all the information relating to flashes, intensity, number of episodes, affected area. In the study group patients, no staining was detected with the starch-iodine test 60 days after treatment at the BoNT-A-treated sites. Six months after treatment, patients reported a relapse of excessive sweating, but with less severe symptoms than baseline. In the control group no significant differences in the mean intensity of sweating or in the mean number of hot flashes were noticed. After the 180 days follow-up symptoms gradually returned to pretreatment levels. Beyond the apparently satisfactory results, the authors recognized some limitations of the study: The difficulty in detecting and treating the entire skin area exactly affected by hot flashes; the difficulty for patients treated with BoNT-A to experience redness because sweating was less or absent. In 2013 Geddoa et al. [64] conducted an uncontrolled single-arm study of 22 patients with primary hot flashes. The affected areas to be treated with BoNT-A injections were neck and chest: Each area was divided into squares of 1 cm and 2 U of onabotulinic toxin were injected intracutaneously for each square for a maximum of 100 U. The DLQI (Dermatology Life Quality Index) questionnaire was assessed at baseline and at the follow up. Four weeks after treatment, the quality of life was significantly improved. In details 20 patients out of 22 (90.9%) reported immediate improvement with almost complete resolution of their flushing, while the remaining two patients needed a re-treatment to achieve the same results. Botulinum toxin appears to be an effective therapy not only for primary or postmenopausal flushing but also in refractory erythema and flushing in patients with rosacea, as demonstrated in a report of two cases by Park et al. [65] and in a literature review conducted by Abokwidir et al. [66] Some studies have recently been published demonstrating the effectiveness of combining laser and botulinum toxin injections to improve erythema and flushing in patients with rosacea. Al-Niaimi et al. [67] experienced the successfully combination of both pulsed dye laser and intradermal botulinum toxin type-A in erythema and flushing in 20 rosacea patients. They measured the degree of erythema using a 3D Anthera camera in order to quantify the results. They demonstrated high efficacy and satisfaction rate with this combined approach and a low side-effect profile. Moreover Friedman et al. [68] conducted a retrospective review of 16 patients aged 23–45 years with Fitzpatrick Skin Types II to IV and facial erythematotelangiectatic rosacea treated by Tixel followed by topical application of 100 U of abobotulinumtoxin. The Mexameter, the Clinicians Erythema Assessment (CEA), Patients self-assessment (PSA) scores, and the dermatology life quality index (DLQI) were assessed at baseline and 1, 3, and 6 months after the last treatment. The scores of the assessments were significantly improved after 6 months after the last treatment compared with baseline (all had a *p*-value < 0.001). All photographs taken with standardized high-definition digital camera photographs, documented the flushing and erythema improvement. Self-rated patient satisfaction was high without side-effects.

The available data on the use of BoNT-A in these diseases is still questionable and published studies have revealed conflicting results in terms of efficacy. Furthermore, it is difficult to objectively assess transient and subjective signs such as erythema and flushing. Not least, the studies conducted up to date have a very short follow-up with minimal long-term data on efficacy and safety [69,70,71]. Table 2 resumes published studies concerning flushing and erythema treated with botulinum toxin.

### 2.3. Raynaud Phenomenon

Raynaud phenomenon (RP) is defined as a medical condition in which spasm of small vessels cause episodes of reduced blood flow in the extremities in response to cold and emotional stress. Three classic phases of color change are described when RP occurs: From pale (vasoconstriction) then cyanotic (ischemic phase) to ultimately red (reactive hyperemia) [72]. RP can be a primary and isolated disease or it can be an onset sign of systemic sclerosis (SSc). It is defined as secondary RP if it is associated with another medical condition (secondary RP) [72]. Primary RP disease can be controlled thanks to simple precautions in daily behaviors such as avoiding colds, minimizing stress, discontinuation of smoking and caffeine intaking, and avoiding vasoconstrictive drugs [73]. RP is usually treated with dihydropyridine calcium channel blockers as first line agents, other treatment options may be topical glyceryl trinitrate, phosphodiesterase 5 inhibitors, a prostacyclin analogue (iloprost), an endothelin receptor antagonist (bosentan), or surgical sympathectomy [72,74]. Botulinum neurotoxin type A (BoNT-A) is emerging as a therapeutic resource for Raynaud’s phenomenon (RP) [73]. However, the mechanism of BoNT-A in antagonizing the constriction of arteriola in RP remains unclear. Zhou et al. [75] basing on the rat cremaster model, showed that BoNT-A could significantly inhibit electrical stimulation-induced arteriole vasoconstriction through the sympathetic pathway. The final result was impaired vesicle fusion with the presynaptic membrane after BoNT/A treatment, inhibiting the release of the noradrenaline. A pilot study published in 2004 was the first report of the possible therapeutic use of BoNT-A in 2 patients affected by RP [76]. In 2007, Van Beek et al. [77] reported the results of treatment of 11 patients with vasospasm associated with a connective tissue diseases. After these encouraging findings, Neumeister et al. [78] published a retrospective review on 19 Raynaud patients injected with BoNT-A for treatment of ischemic pain of hand digits. Fifty to 100 U of BoNT-A were injected into the palm around the neurovascular bundles at the metacarpophalangeal joint of each hand with a dilution of each 100 U of BoNT-A in 20 mL of physiological saline solution; Sixteen of the 19 patients reported rapid resolution of pain and, among these, 13 reported instantaneous improvement; the other 3 patients reported a more gradual reduction over the following 1–2 months. Finally, regarding chronic finger ulcers, these healed within 60 days after treatment. A 3 years retrospective study, involving 15 patients with severe Raynaud’s phenomenon, treated with infiltration of 100 botulinum toxin units type A in 5 mL of saline serum to 0.9% (dilution: 20 IU/mL) was more recently conducted by Medina et al. [79]. Data were recorded at baseline and patients were later evaluated at 30 min, 7 days, 1 month, 3 months, 6 months, and a year from the injection. The follow-up periods were different among patients and more in details: 3 years for six patients, 2 years for four patients, and 1 year for four patients. The patients’ overall level of satisfaction was registered at the end of the data collection period with scores ranging between 0 (very unsatisfied) and 10 (totally satisfied). A statistically significant reduction in pain from baseline was reached, as well as a decrease in the number of weekly episodes of Raynaud’s phenomenon. Of the seven patients with basal ulcers, five were completely resolved at 3 months. Of the patients, 64.3% showed an high overall satisfaction level, with a score >8 at the end of treatment. No serious adverse events were noticed.

Since 2006, several cases reports are now published in literature [80,81,82,83,84]. Winter et al. [83] published a case series of four patients with secondary RP with underlying conditions of rheumatoid arthritis, systemic lupus erythematosus, Sjogren’s syndrome, and scleroderma successfully treated with abobotulinum Toxin A. Two patients reported improvements persisting over a 12-month period with only a single treatment of 300 units of abobotulinum toxin. The remaining two patients reported similar results but required more injections to treat intermittent exacerbations in their fingers and toes. None of the four patients approached the maximum recommended dose of 1000 units for abobotulinum toxin A, not even those with bilateral hand and foot RP. Berk-Krauss et al. [82] described improved pain and discomfort with manipulation of the digit in a 75 years old female patient suffering from CREST syndrome, multi-failure to the systemic previous therapy.

A single-center prospective study [84] was conducted to evaluate the efficacy of botulinum toxin A on younger than 18-year-old patients with primary and secondary RP. Authors enrolled 8 patients aged between 14 and 17 years. BoNT-A was injected into each hand without sedation or anesthetic blockade. The primary outcome was pain reduction after BoNT-A injection and 7 of 8 patients reached the goal. Pain intensity was evaluated at baseline and in the first follow-up. Secondary outcomes included variations in the number and severity of RP episodes after the BoNT-A injection. Among the 7 patients there was a trend towards a reduction in the frequency of RP episodes. One patient did not feel any changes. No patients reported any loss of strength in the thumb or index finger.

Two randomized controlled trials studies on BoNT-A use in RP are available: The first was undertaken by Jenkins et al. [85]. The authors randomized 10 patients to receive BoNT-A injections into one hand, while the contralateral hand was injected with saline as a control They evaluated the change digital pulp temperature as primary outcome of the study. A significant increase in digital pulp temperatures of the hands treated with BoNT-A was registered after 6 weeks from the treatment from baseline as compared with the control hands. Later Bello et al. [86] published a randomized, double-blind, placebo-controlled clinical trial, conducted on 40 subjects with scleroderma-associated RP who received BoNT-A (50 units in 2.5 mL) in one randomly selected hand and sterile saline (2.5 mL) in the contralateral hand. Primary outcome was the change in blood flow detected using the Moor LDI2-IR scanner, from baseline to 1-month follow-up. A statistically significant difference in reduction in average blood flow was noticed in BoNT-A hands compared to placebo 4 weeks after treatment, while change in blood flow at 4-month follow-up was not significantly different between groups. Fregene et al. [87] made a comparison of the different injection sites (wrist, neurovascular bundles of digits, and the distal part of the metacarpus) but they found no significant difference in terms of efficacy outcome. Some authors have discussed the technique for performing BoNT-A injections [88]. Different injection sites have been approached, mainly from the palm of the hand, including digital injections along the neurovascular bundle, in the distal palm near the superficial palmar arch and web space, and in the proximal hand at the distal volar wrist crease adjacent to the radial and ulnar arteries. A palmar injection approach leaves the lumbrical muscles vulnerable to the BoNT-A and transient paralysis could occur. In addiction Dhaliwal et al. [89] proposed a dorsal approach to avoid the side effects of a palmar approach. They experienced the dorsal approach around the digital neurovascular bundles of all five digits in 40 female patients with secondary RP. All patients were treated with a total of 100 units of BoNT-A across both hands (Botox; Allergan, Marlow, UK) reconstituted with 2 mL of normal saline by a single surgeon. After 6 weeks from treatment, 80% of the patients reported a significative improvement in terms of pain, color change, and swelling. The improvement was maintained in the 70% of patients at week 12. No patients referred hand weakness as side effect. Pain and itching in the site injection were reported by two patients, but these symptoms fully resolved in few days. Dorsal approach seems to be effective and safe, although randomized controlled trials are needed to standardize the injection technique.

In some published studies, it is not well distinguished in enrolled subjects whether they were identified in primary or secondary RP. In the prospective case series study by Motegi et al. [90] 10 patients affected by RP concomitant to SSc were enrolled and treated with BoNT-A.

There is not enough evidence to appraise the efficacy of BoNT-A in Raynaud’s phenomenon. Despite many promising reports, further research in the form of randomized controlled trials is needed in order to investigate this new treatment method for Raynaud’s phenomenon [91].

Considering all the studies summarized in Table 3, only few patients were enrolled and there is not even a unique standardized protocol for injections or for outcome evaluation. In addition, some authors have not well distinguished the coexistence of an SSc or other diseases with the RP, creating a bias in the evaluation of BoNT-A efficacy [73,92,93].

Finally, a prospective, single-blind (patients-blind), randomized trial at a single center in Japan was conducted to evaluate efficacy and safety of botulinum toxin B (BoNT-B) for treatment of Raynaud’s phenomenon and digital ulcers (DUs) in patients with systemic sclerosis [94]. A total of 45 patients with systemic sclerosis and secondary Raynaud’s phenomenon were enrolled and randomly divided into 4 groups: A no-treatment control group, and 3 treatment groups, consisting in 250, 1000, or 2000 international units (U) of BoNT-B injections in the hand with more severe disease. The authors demonstrated that 1000 and 2000 U of BoNT-B injection per hand could have a good therapeutic effect on RP and RP-related DU in patients with SSc without any serious adverse events related. The duration of clinical efficacy of BoNT-B was sustained for 16 weeks after a single injection. The limitation of this study is the lack of placebo control group.

### 2.4. Pompholyx

Pompholyx or dyshidrotic eczema is a very common disease which affects the palm and/or soles, characterized by the presence of vescicular-bullus lesions with a chronic-relapsing course. The etiopathogenesis of this condition is not well known, but it is currently considered as a possible manifestation of atopy or contact dermatitis [97]. The most important trigger factors are wet works, sweating, and occlusion [98]. Patients refer pain, itching, burning sensation, and great discomfort in wearing gloves or shoes; moreover bacterial and fungal infections can overlap and complicate the clinical condition. Nowadays several topical, systemic and physical treatments are available [99]. In 2002 Swartling et al. [97] described first an improvement in hand eczema in patients treated with BoNT-A for palmar hyperhidrosis. They published the results of their trial including 10 patients with bilateral dyshidrotic hand dermatitis; one hand was treated with BoNT-A injections (100 U Botox^®^ diluted in 1 mL of saline), while the contralateral was not treated and used as a control at the follow-up. In 7 of 10 patients a good or very good effect of the treatment was noticed. Klein et al. [100] replied to this article with a letter remarking the concept that the therapeutic action of BoNT-A in hand eczema was probably not simply limited to the reduction of hyperhidrosis. They proposed alternative mechanisms to explain therapeutic results. BoNT-A may inhibit the release of neurotransmitters and neuropeptides other than acetylcholine including substance P and calcitonin gene-related peptide. Furthermore, BoNT-A may have a direct effect on afferent fibers, suggesting an inhibition potential of the sensory system. These mechanisms could justify the use of BoTN-A in palmar eczema not associated with hyperhidrosis, such as in palmar eczema associated with atopy. Wollina and Karamfilov [101] performed a prospective side-by-side controlled clinical pilot study using topical corticosteroids (TCS) in association with intracutaneous injections of 100 U of BoNT-A (Botox^®^, diluted in 2 mL of saline). They enrolled 6 patients and they treated both hands with TCS and the more severely affected hand with the association of BoNT-A injection. The authors observed a rapid improvement in pruritus and vesiculation in the hand treated with combination therapy. They sustained that the therapeutic effect of BoNT-A in pompholyx in not only explained by its anhidrotic effect, but it is also due its inhibition of substance P. In 2007, other 2 cases of palmar pompholyx improved after BoNT-A treatment for palmar hyperhidrosis are described [102]. Ismail et al. [103] focused their attention on chronic dry palmar eczema. They conducted a prospective non-randomized side-by-side comparative study involving 30 cases of chronic bilateral dry palmar eczema without hyperhidrosis. They compared, in terms of efficacy and tolerability, combined emollients and topical mild-potency steroid treatment on one hand with an association therapy of 100 units of intradermal BoNT-A on the opposite hand, using both patient- and physician-oriented scores over a period of 6 months. Both lines of treatment were effective and well tolerated by the subjects but a significantly greater improvement of symptom and sign and higher overall patient satisfaction on the hand receiving content-type="color:#505050">BoNT-A were noticed. Moreover, the duration of the effects lasted for a significantly longer period in the side treated with BoNT-A (4 months) as compared with the other side (1 month). To date, there are no studies on the therapeutic use of BoNT-A in plantar eczema in the literature. This is probably due to the same reasons that limit its use in plantar hyperhidrosis (pain, extension of the plantar area, need for more units and related higher cost, and lower effectiveness). In addition, the dilutions would not be the same as those used in hyperhidrosis and this is not well explained. Placebo-controlled trials are currently missing. The studies are resumed in Table 4.

### 2.5. Eccrine Nevus

Congenital eccrine nevus (EN) is defined as a rather rare cutaneous hamartoma that is recognized in histology by the increase in the number and/or size of eccrine glands, not associated with vascular proliferation. The absence of vascular elements distinguishes EN from other conditions such as angiomatous eccrine hamartoma [104]. EN is predominantly localized in forearms without underlying skin alterations except a localized area of hyperhidrosis [105]. Therapeutic management is conditioned by the area of extension and the severity of hyperhidrosis: Topical agents or surgical excision is the most common options. Treatment with botulinum toxin represents an additional therapeutic resource of which only a few case reports are published in the literature. Honeyman et al. [106] reported a case of congenital EN of the right wrist in a 12-year-old girl resistant to topical antiperspirant agents; since hyperhidrosis limited social and daily activities and surgical excision was not possible due to the size of the lesion and the sensitive anatomical site, the patient was treated with BoNT-A.

Five U per point, at intervals of 0.5–1 cm. of BoNT-A was injected. The BoNT-A was diluted in 4 mL of saline solution 0.9%, but the authors did not specify the total amount of toxin nor the timing of the first response to BoNT-A treatment. They described the 1 year follow up evaluation reporting a significant decrease in sweat episodes to once a month and the improvement in patient’s quality of life. In 2015, Lera et al. [104] experienced the treatment with BoNT-A in a patient with EN on the forearm; at baseline, the hyperhidrosis disease severity scale (HDSS) score was 3 (severe) and the patient referred a poor quality of life. BoNT-A was reconstituted with 2.5 mL of 0.9% sterile saline solution and, after the Minor test has been performed to highlight the area to be treated, 2 U per site were injected (a total of 100 IU). Forty-eight hours after treatment the patient reported a decrease in sweating with maximum response at week 3, achieving a score of 1 on HDDS (mild hyperhidrosis). After 9 months, the BoNT-A treatment was repeated due to the recurrence of hyperhidrosis. Sonntag et al. [107] published a case report on a 22 years old patient with EN on the back of right hand, associated to a localized hyperhidrosis causing great discomfort in daily activities (writing, shaking hands). She was treated with one the intradermal injection, of 200 mU Dysport^®^ in total. The maximum anhidrotic effect was maintained until the week 30 after the treatment, then sweating relapsed, and 4–6 weeks later patient worsened to a condition requiring a new treatment. BoNT-A injection therapy has proved to be effective also in the eccrine angiomatous hamartoma [108]. Despite the rarity of EN, in selected cases, botulinum toxin represents a valid therapeutic option for patients suffering from this condition. Table 5 describes 4 cases reported in the literature.

### 2.6. Postherpetic Neuralgia

Postherpetic neuralgia is the most common and very difficult to treat complication of herpes zoster, characterized by the persistence of chronic and debilitating pain. Pharmacological therapies are multiples, including non-steroidal anti-inflammatory drugs, gabapentin, opioids, and tricyclic antidepressants, as well as topical anesthetics [109], but pain can be resistant to all of these approaches. In 2010, Xiao et al. [110] conducted an interesting randomized controlled clinical trial on 60 subjects suffering from postherpetic neuralgia, investigating the analgesic effect of BoNT-A injection with lidocaine compared to placebo (saline). In the treated group, patients reported a decrease in VAS pain more significant than in the control groups. Li et al. [111] conducted a systematic review and meta-analysis to evaluate the safety and efficacy of local administration of BoNT-A vs. lidocaine in the treatment of post-herpetic neuralgia. Among the 570 articles identified from the literature search the authors eventually included 7 RCTs in the meta-analysis, involving 752 subjects (367 subjects in the BoNT-A group and 385 subjects in the lidocaine group) monitored for a period of 3 months after treatment. BoNT-A (total dose, ≤100 units) was administered by subcutaneous injections in the area where herpetic lesions had previously arisen and where pain was currently located, at the proximal end of the nerve branch in the damaged tissue. As primary outcomes the authors evaluated improvement in Visual Analogue Scale (VAS) pain scores at 1, 2, and 3 months after treatment and the effective rate. Secondary end-points were scores on the McGill pain questionnaire and adverse event rate. The results of the meta-analysis showed a significantly improvement in VAS pain score, achieving lower scores at the follow up assessments, a significantly higher effective rate and an improvement of the scores on the McGill pain questionnaire in patients who received BoNT-A for post-herpetic neuralgia compared to those who received lidocaine.

Wei et al. [112] also conducted a meta-analysis to synthesize existing evidence for the management of trigeminal neuralgia (TN) and peripheral neuropathic pain (PNP), with BoNT-A. They included 10 RCTs with a total of 391 patients. Routes of administration for BoNT-A injection included subcutaneous, submucosal, or intradermal while the dosage of ranged from 25 U to 300 U of BoNT-A. The pooled data of meta-analysis showed that BoNT-A offers significant benefit in the treatment of patients with TN and PNP compared with placebo, increasing the percentage of respondents’ subjects and improving pain scores at follow-up. The two reported meta-analyses both show some limitations such as the small size of patients enrolled in the clinical trials. Moreover, the significant differences in the baseline characteristics of the patients of both treatment groups, included in several of these trials represent a potential source of heterogeneity. Large well-designed RCTs are needed to validate this conclusion.

A third meta-analysis was conducted to evaluate the efficacy of the use of BoNT-A to treat trigeminal neuralgia and postherpetic neuralgia. Six double-blinded, randomized, placebo-controlled studies were included. The author concluded that patients treated with BoNT-A injection were about 2.9 times more likely to have a 50% or more reduction in pain compared with the placebo group. The overall strength of the evidence was moderate because of the small number of studies and risk of bias.

Jain et al. [113] published the interesting cases reports of two pregnant women aged 36 years and 38 years, respectively, who developed Herpes zoster in 28 and 32 weeks of gestation. Both the patients were treated symptomatically, and acyclovir was not administered in either of the cases. As the, pain persisted even after 4 weeks in spite of analgesic and antiepileptic treatment, BoNT-A in fixed dose of 500 units Dysport was administered. The toxin was diluted with 5 mL of normal saline, making a concentration of 100 units/mL. A significantly reduction in pain was noticed after treatment and both the patients remained comfortable during the pregnancy period. Both the babies delivered were normal and are growing up normally.

Single centers studies [114,115] reported real life experiences about the efficacy of intradermal injection of botulinum toxin in patients suffering from PHN. They differed in the size of the sample, timing and dosing of the treatment. The authors sustained that botulinum toxin treatment is effective in reducing pain in PHN and the decrease is less prominent across time.

From the cited studies, we noticed that the routes of administration of BoNT-A in the treatment of PHN are multiple: Subcutaneously [111], intradermally [116], with a chessboard [117] or fanning [118] pattern and under ultrasound guidance [119]. The injection of botulinum toxin could play a role in the reduction of various substances that sensitize nociceptors [117], and thanks to its analgesic potential represents one of the most important resources for chronic neuropathic pain (Table 6).

### 2.7. Oily Skin

In 2008, Shah [71] conducted a retrospective analysis on 20 patients in order to investigate the safety profile and subjective efficacy of intradermal BoNT-A in facial (“T-zone”) pore size and sebum production. The main limitation of this study was the lack of an objective measurement of sebum production. The efficacy results are only based to the subjective patients’ satisfaction for the improvement in sebum production and the decrease in pores size. However the encouraging findings of these preliminary data inspired the prospective study of Rose and Goldberg [120]. The aim of their study was to evaluate efficacy and safety of intradermal botulinum toxin for the treatment of oily skin in the forehead region. Twenty five subjects were enrolled; the efficacy was investigated subjectively by the patient with a satisfaction scale and objectively by the physician with sebometric readings at 4 follow-up points assessed at the baseline after treatment. Pre- and post-treatment photographs were also taken. Each 300-U vial of abobotulinum toxin A was diluted using 3 mL of saline solution and was injected intradermally into 10 injection sites of the forehead. Three to 5 U of botulinum toxin were injected at each point (total amount of 30–45 U). The authors reported a significantly lower sebum production, and 91% of patients were satisfied. The mechanism by which intradermal botulinum toxin leads a reduction in sebum production is not entirely understood because the role of the nervous system and acetylcholine on sebaceous glands is not clear. Probably the arrector pili muscles and the local muscarinic receptors in the sebaceous gland are targets to neuromodulatory effects of BoNT. Li et al. [121] showed that human skin sebaceous glands in vivo and sebocytes in vitro express nicotinic acetylcholine receptor α7 (nAchRα7), and that acetylcholine increased lipid synthesis in a dose-dependent manner. They enrolled 20 healthy volunteers in a double-blind, placebo-controlled, split-face trial. Immunohistochemistry and immunocytofluorescence were performed to evaluate cholinergic receptor levels in sebaceous glands. A significative decrease in sebum production on the botulinum-treated side was found after treatment in volunteers with oily skin.

For an effective treatment, the injection technique and placement are crucial to the treatment of oily skin. A procedure that promotes the correct placement into the dermis is inserting the needle at a 75° angle and considering the extrusion of toxin from adjacent pores as an endpoint [71]. Shah et al. [71] through a photographic assessment, documented a reduction in pore size, but this method is not accurate; however, previous studies demonstrated that sebum level strictly correlates with pore size [120]. Further study is needed to define the optimal injection techniques, doses, and applications for oily skin and enlargement of pores.

Two retrospective reviews published in 2017 and 2019 were conducted by Endly et al. [122] and Shuo et al. [123] respectively. While Endly reported data on the entire panorama of therapeutic options for the oil skin, Shuo and colleagues focused only on botulinum toxin treatment. They noticed that most studies have suggested the role of the intradermal injection of BoNT-A in decreasing sebum production and pore size. Furthermore, this treatment resulted in high patient satisfaction without significant side effects. Intradermal BoNT-A injection may represent a innovative promising treatment for oily skin and other relevant dermatological problems, such as enlarged pores, acne, and seborrheic dermatitis. Further studies are still needed to determine the specific mechanisms of BoNT-A and to standardize the optimal injection techniques and doses for oily skin and other relevant cosmetic concerns.

### 2.8. Notalgia Paresthetica

Notalgia paresthetica (NP) is a sensory mononeuropathy of unknown origin and that usually affects the skin of the dorsal segments D2–D6 which represents a therapeutic challenge and that needs to be treated. Patients suffered from pruritus, pain, paresthesia, hypo- and/or hyperesthesia, and burning. NP is clinically defined by a brownish patch and the involved area is constantly scratched; NP mainly occurs in older patients or is often linked with musculoskeletal compression of spinal nerves [124]. The first 2 cases of NP treated with botulinum toxin were published in 2007 [125]. In 2010, Wallengren and Bartosik [126] experienced and described the botulinum toxin treatment on 6 patients affected by NP or neuropathic pruritus with a small improvement of symptoms. Later, Perez et al. [57] described a cases series of five patients diagnosed with NP. Previous treatments had failed and none had achieved resolution of itching. The 5 patients have been treated with intradermal botulinum toxin A; every vial of BoNT-A was reconstituted with 2.5 mL of normal saline (0.9%) and an insulin syringe was then used to inject 4 units (0.1 mL) at each injection point. The total dose received by each patient ranged from 48 to 56 UI and it was determined by the size of the affected area. The authors evaluated severity of itching using a visual scale numbered from 0 to 10 at baseline and 1, 6, 12, and 18 months after treatment. Variable results were observed after the administration of intradermal botulinum toxin. None of the patients obtained the complete resolution of the pruritus. Three patients improved temporarily itching (1 month) and 2 patients worsened after treatment. Only 1 double- blind randomized clinical trial concerning the use of BoNT-A as a therapy for NP is currently available in literature [127]. This study was published in 2014 by Maari et al. They enrolled 20 patients affected by NP, resistant to previous topical therapies; patients were randomized to either BoNT-A (onabotulinum toxin A) or saline solution alone (placebo). The area to be treated was defined by the presence of hyperpigmentation, otherwise if the hyperpigmentation was absent the same patient defined the region limited to the itching area. Patients in the botulinum toxin arm received injections of 0.1 mL (50 U/mL) for every 1–2 cm^2^ (maximum intradermal dose of 200 U BoNT-A). Patients in the placebo group received the corresponding volume of saline solution. All patients continued the study until week 24; subjects who received placebo at baseline received BoNT-A at week 12. VAS scale for pruritus was assessed to evaluate the improvement in pruritus from baseline to the end of observation period. Eight weeks after treatment statistical analysis revealed no significant mean difference in pruritus VAS between patients treated with BoNT-A and those that received placebo. There was no significant difference between the two groups also considering hyperpigmentation or investigator and patient global efficacy assessment. Recently Ansari et al. [128] published a review with an algorithmic approach for the treatment of notalgia parestesica. They suggested that treatment should start with topical agents or physical therapy, then systemic agents, and finally procedural modalities such as toxin botulinum injections. They recommended combining treatment options with physical therapy to maintain a treatment response by the time. Table 7 collects all the cases previously described.

### 2.9. Hailey–Hailey Disease

Hailey–Hailey disease (HHD) or familial benign pemphigus is a chronic autosomal dominant acantholytic dermatosis typically characterized by blistering flaccid and erosive lesions mostly affecting intertriginous regions of the skin. This disorder is exacerbated by heat, sweat, and bacterial colonization [129]. These local factors can intensify the reduction of keratinocyte cohesion and therefore the occurrence of lesions on the intertriginous areas, mainly axillary and inguinal folds [130,131]. Treatment is aimed at reaching complete or partial remission as long as possible. The following have been described: Topical, intralesional, and oral steroids, cyclosporine, methotrexate, antimicrobial agents, retinoids, tacrolimus, phototherapy, and other treatments with anecdotal evidence. Surgical strategies include carbon dioxide laser ablation, cryotherapy, dermabrasion, electrosurgery, excision, and grafting [129]. The first successful botulinum toxin (BoNT-A) application in a patient with HHD after a series of failures of conventional treatment was introduced by Lapiere et al. [132] in 2000. They first treated only the axillary folds, first only the left axilla with 25 U, and after 6 months with 50 U in each axilla; they noted no worsening or improvement in the groin. Authors suggested that the effectiveness of BoNT-A injections was linked to a decrease in sweat production and consequently the moisture that triggered microbial growth. Konrad [133] first directly compared BoNT-A treatment with ablation therapy (erbium: YAG laser). They injected BoNT-A on both parts of the sub-mammary area, and after 4 days used dermabrasion or erbium: YAG laser in a limited area of 25 cm^2^ on each side. They showed that BTA alone was effective in inducing remission of HHD. In 2002, Kang [130] confirmed the usefulness of BoNT-A in a recalcitrant HHD case; they treated both the inguinal and axillary folds with a 6-month remission. In a study of six patients, Koeyers et al. in 2008 introduced BTA as a safe and effective adjuvant treatment for extensive HHD [134]. A total of three patients were treated by Lopez-Ferrer and Alomar with different regimens and timing of administration [135]. In 2018, Charlton [136] described a severe HHD case with painful, intermittent blistering plaques and fissures in his axillae and groin which was treated with topical and oral formulations of antibiotics, antifungals, corticosteroids and carbon dioxide laser therapy without benefit. 100 U BoNT-A was administrated in his axillae and groin (50 U at each site) once a year for two consecutive years with a consequent reduction of symptoms to two episodes a year. Recently, Kothapalli [137] suggested botulinum toxin injection be considered as a life-changing first-line treatment for Hailey–Hailey. Table 8 collects all the studies on the use of BoNT-A therapy in HHD.

### 2.10. Genodermatoses

#### 2.10.1. Epidermolysis Bullosa Simplex, Weber–Cockayne Type

Epidermolysis bullosa simplex of the Weber–Cockayne type (EBS-WC) results from a genetic mutation in keratin intermediate filaments 5 and 14 in the basal layer of epidermis that leads to a recurrent blistering rash following frictional trauma, particularly on the hands and feet [138]. In summer and hot climates, patients experience exacerbation of the disease, probably caused by hyperhidrosis; these findings supported 5 double-blind placebo-controlled crossover studies in which different treatments, both topical and systemic, have been tested and failed to show any difference between the treated and placebo groups [139]. In 2010, Swartling [138] performed a retrospective evaluation of the effects of BoNT-A injections in 14 patients with EBS and congenital pachyonychia with foot blisters and painful callosities. The author has performed 1 to 4 treatments with 170–200 U of toxin in each foot with excellent results on symptomatology and limited side effects. More recently Holahan described the use of botulinum toxin to treat plantar blistering and pain in a child (50 U per foot), with excellent but transient (3 months) success [140].

#### 2.10.2. Darier Disease

In 2007, the first reported use of BoNT-A as adjuvant therapy in Darier’s disease occurred when Kontochristopoulos et al. [141] treated with success the submammary areas of a 59-year-old patient. A further case in 2008 also confirmed the usefulness of decreasing sweating in the intertriginous area in a young patient with serious involvement of the non-genital area [142]. She received acitretin 10 mg per day and antimicrobial and antifungal medications for the coexisting infection, but her overall poor quality of life and discomfort remained significant. BoNT-A injections (40 U in each groin crease and 20 U in each anal crease) were administered with significant improvement after 3 weeks in both symptoms and clinical lesions.

A search of the ClinicalTrials.gov website, (a registry/results database of publicly and privately supported clinical trials conducted around the world), revealed the study NCT02782702, started in September 2015 in which the efficacy of the botulinum toxin type A was evaluated in 30 patients suffering from Darier disease. The results of this study are currently not available [143].

#### 2.10.3. Pachyonychia Congenita

Pachyonychia congenita (PC) is a rare autosomal dominant genodermatosis that is mainly characterized by plantar keratoderma, nail dystrophy, and plantar pain. Elevated ambient temperature, summer, and sweating aggravate this condition until disability [144]. In 2006, following this evidence, Swartling and Vahlquist [145] administered BoNT-A in 3 PC patients; the authors also reported not only an anhydrotic effect but also a great improvement in pain and discomfort since acitretin therapy was discontinued. One patient abandoned wheelchair use. The promising experience of Swartling and Vahlquist prompted the same authors to an intriguing retrospective evaluation of the effects of BoNT-A injections in 14 EBS and PC patients with foot blisters and painful callosities [138]. Similar results in blistering and pain improvement were noted; the hypothesis that explains these effects is that BoNT-A may affect nociceptive C-fibers in the epidermis through blocking neuropeptide release from sensory nerve axons and also inhibits neurogenic inflammation. In 2016, 2 new cases of PC treated with BoNT-A injection were reported [146]. Both patients showed a marked improvement in pain and blistering with an average response time of one week, a six-month mean duration of effectiveness, and a lack of any side effects or tachyphylaxis. Recently Koren et al. have reported their 4 years’ experience in using botulinum toxin injections in the treatment of PC-associated keratoderma [147]. Using a structured approach, which includes the use of a sufficient dose of BoNT-A (200–400 U of onabotulinumtoxinA or 500–1000 U of abobotulinumtoxinA), and regular intervals between treatment sessions (of <100 days), the authors have demonstrated the effectiveness of the treatment, with major change in patients’ quality of life.

### 2.11. Hidradenitis Suppurativa

Hidradenitis suppurativa (HS; or acne inversa or Verneuil’s disease) is an inflammatory and debilitating skin disorder with multiple flare-ups. It affects apocrine regions with bullae, sinus tracts, fistulas, and cicatrices. HS patients have severe psychosocial distress and costs [148,149]. Even if HS may not be primarily a disease affecting the apocrine glands, the glands remain a potential therapeutic target [150]. In 2005, the first case of successful use of BoNT-A was described in a young woman with axillary HS with 10 months of complete absence of symptoms [151]. In 2009, Feito-Rodriguez et al. [152] also reported the case of a 7-year-old girl suffering from HS recalcitrant to all topical and systemic medications, with partial remission and early relapse after discontinuation of any therapy. 40 U of BoNT-A on each side were injected with complete remission for 6 months. The reoccurrence responded to a similar second treatment. The efficacy of BoNT-A treatment was confirmed by Khoo and Burova, who used the toxin on 3 patients [153]. One of them, a 46-year-old woman, had Hurley stage II HS and hyperhidrosis involving axillae and groin, unresponsive to conventional therapies already treated with surgical drainage of the abscesses. Over the course of 3 years, he received 4 treatments with 50 U of BoNT-A (100 U dissolved in 4 mL of 0.9% NaCl solution) each time, administered to each axilla. She responded optimally within 3 months of the first treatment, and after the second treatment, complete remission was observed. More recently, several case reports and case series have been published on this topic. Shi et al. [154] have described the use of BoNT-A in a 41-year-old obese Native American woman affected by a HS localized in the axillary and groin regions (Hurley stage III) resistant to all available therapies. She received an injection every 3 months (four times thus far), which had significantly helped alleviate her pain and curbed the progress of her HS by resolving abscesses and healing draining sinuses. Campanati et al. [155] investigated the potential therapeutic role of BoNT-A in HS by treating 2 patients. The first patient, a 23-year-old woman, suffering from axilliary HS, was treated with 50 U of BoNT-A per axilla while the second patient, a man of 50 -year-old with a HS of the groin and inner thighs, was treated with 100 U of BoNT-A for each side. Both patients experienced a very good response.

The exact mechanism by which BoNT-A demonstrates efficacy in HS is unclear. It is known that at skin folds such as the armpits and groin, there are ideal conditions for bacteria to flourish and this is a precipitating factor of HS. The effect of BoNT-A on sweat production can reduce the population of skin flora and its potential proinflammatory effect. Furthermore, if in the past HS was considered primarily a disorder of the apocrine sweat glands, recent studies have shown that we must consider the condition as a disorder of the follicular epithelium [155]. Indeed, an additional hypothesis about the therapeutic effect of BoNT-A is that it prevents the rupture and diffusion of follicular material through the dermis, which usually promotes inflammation and sinus tract formation [151].

Recently, the Grimstad et al. conducted a randomised, double-blind, placebo-controlled pilot study evaluating the effectiveness of BoNT-B in 20 patients with HS. Patients were treated with variable dosages of BoNT-B, depending on the location of the disease, limited per field at up to 150 U/armpit, 200 U/groin, and 600 U in the perianal/perigenital areas. The maximum total dose allowed per treatment was 4000 U of BoNT-B. The authors demonstrated an improvement of DLQI from a median of 17 at baseline to 8 at 3 months in the BoNT-B group, compared with a reduction from 13.5 to 11 in the placebo group (*p* < 0.05). Improvement of the patients’ own ratings of symptoms and a reduction in total lesions supplemented the primary outcome. Fifty-five percent of the study population reported some degree of hyperhidrosis. Table 9 collects all the studies conducted on the BoNT-B application in HS.

### 2.12. Aquagenic Keratoderma

Aquagenic keratoderma (AKD) is a rare skin disease characterized by transient wrinkling of the skin, edema, formation of whitish papules, pruritus, burning sensation, or pain, on the palms and/or soles in response to contact with water [157]. Several cases of AKD treated with BoNT-A injections have been published.

The first one, in 2005, described a 35-year-old woman who presented with a 2-year history of discomfort in her hands after 5 min of contact with water [158]. She also suffered from axillary and palmar hyperhidrosis and after unsuccessful treatment with aluminum chloride and additional involvement of the feet, she was treated with BoNT-A on her palms with great improvement lasting 5 months. Other 2 cases, both published in 2010, demonstrated a good response to BoNT-A therapy [159,160]; Bagazgoitia et al. proposed that this good response was probably due to an involvement of eccrine and sweat glands in the pathogenesis of AKD. Similarly, Montoya [161] suggests that the disorder is caused by aquaporin dysfunction. Recently, two more cases of AKD treated with BoNT-A have been published. In both cases 100 U of toxin was used for each palm of the hand with excellent results [157,162]. In addition, in Rodríguez-Villa Lario ‘s case report, the patient also had a rare dorsal involvement, also improved with BoNT-A.

### 2.13. Alopecia

#### 2.13.1. Alopecia Areata

Alopecia areata (AA) is a common dermatosis that causes patches of nonscarring hair loss on the scalp or eyebrows, beard, and less frequently on other skin areas [163]. The pathogenesis of AA is not fully understood, although strong direct and indirect evidence supports an autoimmune etiology for AA [163]; in 1994, Paus et al. [164] demonstrated that substance P (SP) may play a role in the neural control of hair growth. They suggested an interesting interaction in epithelial-mesenchymal-neuroectodermal system of the hair follicle. In fact, it has been hypothesized that sensory neurons are able to produce a series of neuropeptides of which substances P (SP) and calcitonin gene-related peptide (CGRP), that are able to directly or indirectly modulate hair growth [165]. Therefore BoNT-A may also prevent the release of pain-stimulating neuropeptides in peripheral nerves and affects soluble N-ethylmaleimide-sensitive factor activating protein receptor proteins by decreasing the release of pain mediators, including SP, CGRP, and glutamate.19 This may be accomplished directly by blocking SP from sensory afferent terminals, blocking the release of CGRP from autonomic vascular nerve terminals, or both [165].

The first case of alopecia cured with BoNT-A injections was a 34-year-old woman affected by a cephalalgic alopecia, a rare headache syndrome in which recurrent, burning head and neck pain is associated with hair loss from areas of scalp affected by the pain [166]. The patient failed all previous therapies with increasing and debilitating pain; therefore BoNT-A was injected into procerus, corrugator, frontalis, temporalis, splenius capitus, occipitalis, and trapezius muscles (100 U in total). The beneficial effect, which began 10 days after injection, lasted for 6 weeks, and a second treatment with the same doses was given 3 months later. The patient reported complete disappearance of pain for 60 days and had significant hair regrowth. An additional treatment with BoNT-A was performed after 8 weeks with similar efficacy. Cutrer et al. [166] described other 3 similar cases in 2010. Irimia et al. [167] in 2013 confirmed the efficacy of BoNT therapy for cephalalgic alopecia with another case treated with onabotulinum toxin A. The efficacy of BoNT-A treatment would suggest, in the authors’ opinion, a common pathophysiology for both phenomena characterizing the pathology. Biopsy specimens taken from areas of the scalp of patients with cephalalgic alopecia showed lymphocytic infiltration around the hair bulb and reduced nerve fiber density, including SP-positive and CGRP-positive fibers. These alterations disappeared after onabotulinum toxin A treatment [166]. In 2010, Cho et al. [168] enrolled 7 patients with AA treated with 10 U of BoNT-A injected intradermally at each site monthly for 3 months. The discouraging results led the authors to assert that BoNT-A therapy did not affect hair growth in AA, but they did not rule out that the use of BoNT-A at the right stage and severity may lead to improvement in AA.

#### 2.13.2. Androgenetic Alopecia

Androgenetic alopecia is the main cause of progressive scalp thinning. Over a lifetime, it affects approximately 80% of the male population and 50% of the female population [169]. In 2010, 50 male subjects (19–57 years old) with Norwood/Hamilton ratings II-IV were enrolled and treated with 150 U of Botox^®^ (5 U per 0.1 mL saline) into the muscles surrounding the scalp, including frontalis, temporalis, periauricular, and occipitalis muscles for a total of 30 injection for each sites. The primary outcome measure was a change in hair count in a fixed 2-cm area and the secondary outcome was hair loss, measured with the count of loose hair on the pillow [170]. Forty subjects completed the study and experienced a statistically significant increase in hair count (18%) at week 48. A similar study with the same results was conducted by Singh in 2017 [171]. Compared to his predecessors, Zhang [172] experimented a lower dosage of BoNT-A for male androgenetic alopecia in an open label study in 2019. Among 24 patients treated with 50 U of toxin, after 6 months of treatment, 11 patients showed significant hair regrowth (>10% from baseline), 8 showed minimal improvement, and 5 had no changes. In addition, grease secretion gradually restored to the normal state in 19 patients who had shown a significant decrease in grease secretion at 3 months and the grease content was close to the healthy level. Most importantly, no patients had any adverse events. The hypothesis supporting the use of BoNT-A in androgenetic alopecia is closely related to the low-oxygen environment in the areas of the scalp most affected by the hair follicle miniaturization phenomenon. Blood flow may therefore be a primary determinant in follicular health, so much so that Goldman et al. [173] have demonstrated the presence of microvascular insufficiency in scalp areas affected by hair loss in male pattern baldness. BoNT-A injected into the scalp would therefore appear to decrease pressure on the perforating vascular system with an increase in blood flow and oxygen concentration. Furthermore, the enzymatic conversion of testosterone to dihydrotestosterone, the actual hormone responsible for hair loss, is oxygen dependent. Thus, where the oxygen rate is low, this conversion is favored, whereas in high-oxygen environments, the conversion is lower resulting in the conversion of testosterone to estradiol by the aromatase enzymes [169]. Recently Shon et al. [174] conducted an open label pilot study with 18 patients suffering from androgenetic alopecia treated with 30 U BoNT-A every 4 weeks for 24 weeks. They also suggested a new pathophysiological mechanism of disease development, arguing that the Dihydrotestosterone (DHT) induces transforming growth factor1 (TGF-Beta) in dermal papilla cells (DPCs) to suppress follicular epithelial cell growth. Other than getting good results after therapy, they found that DHT upregulated the TGF-Beta expression of DPCs in96 h, whereas BoNT-A downregulated the TGF-1expression in 96 h. Finally, Zhou et al. [175] conducted a randomized open label trial in 63 patients with androgenetic alopecia in which the use of the BoNT-A alone and the use of the BoNT-A combined with finasteride were compared. They demonstrated that BTA is a safe and effective therapy for the management of AGA and BTA combined with FNS presents excellent results. It should be noted, however, that the BoNT-A used for the treatment of facial wrinkles has been associated with the appearance of frontal alopecia. Di Pietro and Piraccini conducted an observational study on five females who complained of progressive recession of the hairline after periodic injections of botulinum toxin type A in the forehead [176].

Now a phase IIb clinical trial is ongoing under the approval of Korean Food and Drug Administration; at the end of the trial, will be available more data about botulinum toxin and androgenetic alopecia [174]. Table 10. collects all the studies conducted on the BoNT application in androgenetic alopecia.

### 2.14. Psoriasis

Despite the wide range of treatments available for psoriasis, including biologic therapies [177,178,179] and small molecules [180], scientific research has also moved in other directions. A series of studies have attempted to define the relationships between psoriatic disease and the nervous system by demonstrating a high concentration of nerve fibers in psoriatic skin and an increased level of CGRP and SP of sensory nerve origin. Thus, this hypothesis is supported by the fact that following a loss of innervation, nerve function, or injury to the nervous system, clinical remission of the disease is observed [181]. Zanchi et al. [182] and more recently Aschenbeck [183], demonstrating clinical improvement in inverse psoriasis following BoNT-A injections, hypothesized that the toxin inhibits CGRP- and SP-derived nerve release. Histologic resolution of the condition was demonstrated by Ward et al. [181] who observed, using the adult KC-Tie2 mouse (a mouse model of psoriasiform dermatitis) that intradermal injections of BoNT-A lead to significant improvement over placebo in acanthosis and a reduction in skin lymphocyte infiltration. However, clinical reports and observational study published are few and not placebo-controlled. Zanchi et al. [182] observed clinical improvement in 15 patients with inverse psoriasis treated with BoNT-A but this study had limitations because the results were assessed only by patient self-assessment (VAS scale for itching and pain) and photographic assessment of erythema and infiltration. For this reason, Chroni et al. [184] raised two major criticisms, namely the absence of a quantitative measure to estimate improvement (PASI score, for example) and of histological evaluation before and after treatment. In Saber [185] and Gilbert [186] case reports, similar results are presented, but the cases have the same criticisms.

In these cases, the authors hypothesized that the beneficial effects of BoNT-A resulted from the reduction of local sweating in folds, such as HHD (or chronic benign familial pemphigus) [129,132,187,188] However, patients also referred to a decrease in pain and pruritus, likely due to BoNT-A’s ability to block the release of algogenic neuropeptides.

More recently, other authors, have tried to quantify the improvement produced by the toxin on the single plaque by measuring the Total Clinical Score (TCS) composed of the sum of erythema (0–3), desquamation (0–3), and infiltration (0–3) of every single plaque [189] or by measuring PASI and PGA [183].

Finally, Todberg et al. [95,190] conducted an exploratory, multicenter, randomized double-blinded trial, where it has been evaluated the effectiveness of the BoNT-A on a single plaque versus placebo. The effect was evaluated at 8 weeks and no clinical or histological differences from the vehicle were found. Bagherani et al. [190] in a case series study of 20 patients treated with BoNT-A injected as single therapy, nine injections of 4 Units, 0.04 mL in total in a single plaque, failed to demonstrate improvement of skin lesion. Table 11 schematizes the cases reviewed.

## 3. Discussion

This review emphasizes the great potential of the use of BoNT in a large number of heterogeneous skin diseases. Currently, all molecular and pathophysiological mechanisms underlying the therapeutic effects BoNT are not established yet. It can be inferred that many of the diseases that affect the folds (inverse psoriasis, HHD, HS) can improve after injection of botulinum toxin probably owing to its anhidrotic effect reducing bacterial contamination and maceration. Further studies should be designed to investigate the role of BoNT in neuropeptides regulation and the link with the neuroimmune system in order to better understand its therapeutic potential in several skin diseases.

Taking into account all reported data, we could postulate to draw a gradient of literature evidence of the efficacy of BoNT in skin diseases, as follows: Focal idiopathic hyperhidrosis, facial erythema and flushing, postherpetic neuralgia, notalgia paresthetica, psoriasis, suppurativa hidradenitis, Hailey–Hailey disease, oily skin, epidermolysis bullosa simplex, Darier’s disease, pachyonychia congenita, aquagenic keratoderma, alopecia.

For several skin disease, botulinum toxin has been used in off-label regimen for several chronic skin disease, however several questions still remain open nowadays: For how long BoNT can be used without any risks or sequelae? is tachyphylaxis a plausible event limiting its effectiveness over time? In this regard, it has already been established the immunogenic potential of BoNT, whether these antibodies are neutralizing or not, has to be established yet. Finally, a consensus on the dose regimen for each indication and well-defined injection techniques for standardization of all the therapeutic protocols are desirable.

## 4. Materials and Methods

This systematic review is reported based on the Preferred Reporting Items for Systematic Reviews and Meta-Analysis (PRISMA) guidelines [191].

### 4.1. Literature Searches and Selection

We performed a worldwide systematic review of studies reporting botulinum toxin in dermatology, using 3 electronic medical databases–PubMed, EMBASE, Web of Science.

The search terms were selected to identify studies describing therapeutic profile of botulinum toxin in dermatological setting.

The following search terms have been used: “botulinum toxin”, “dermatology”, “skin diseases”, “focal idiopathic hyperhidrosis”, “Hailey–Hailey disease”, “epidermolysis bullosa simplex Weber–Cockayne type”, “Darier’s disease”, “pachyonychia congenita”, “suppurative hidradenitis”, “aquagenic keratoderma”, “alopecia”, “psoriasis”, “notalgia paresthetica”, “facial erythema and flushing”, “oily skin”, and “Raynaud phenomenon”.

All of these databases were searched from their respective inception dates to the 8 November 2020. In addition, we searched by hand the reference lists of other relevant articles on dermatological use of botulinum toxins. Additionally, government reports, and grey literature available on botulinum toxin and dermatology were searched. No language, regional or temporal restrictions were applied, except for focal idiopathic hyperhidrosis, whose research was limited to the last 10 years.

### 4.2. Data Extraction

The articles retrieved by the search were screened by title and selected for further review if they reported data on botulinum toxins and skin diseases, or dermatological management strategies. Duplicate references were removed. Two independent reviewers (AC and EM) screened the literature search and assessed each study for inclusion. Any disagreement was solved by consulting a senior investigator (AO). Articles meeting the eligibility criteria following review of the full text (English language, clinical trials, randomized and controlled clinical trial, meta-analysis studies, case series) were selected for data extraction. The data were collected according to a form, listing authors, country/geographic region, study population, estimate, and ages of subjects.

## Figures and Tables

**Table 1:** BoNT-A treatment in bromhidrosis.

First Author [Ref.], year	Type of Study	n (site)	BoNT-A Doses	Retreatment	Follow-Up	Results
Wu [54], 2019	Prospective	19 (axilla)	NA	NA	3 months	mean degree of malodor and mean sweat production in the BoNT-A-treated axilla were significantly lower than those in the control axilla at 3 months after therapy.
Wang [55], 2018	Prospective	62 adolescents (axilla)	50 U BoNT-a/axilla	Yes	The average follow-up was 2.64 years	82% of patients (51/62) ranked the BoNT-A treatment to be very good or good.
He [53], 2017	Prospective	53 (secondary bromhidrosis, axillae)	50 U BoNT-a/axilla	NA	12months	48 patients ranked the satisfaction with BoNT-A treatment as “very good” or “good”
Lee [56], 2004	Cae report	1 (genitalia)	40 different sites (2.5 mU/0.1 mL per site)	NA	9 months	odorless and anhydrous response in the genital region,

**Table 2:** Studies on the use of BoNT-A therapy in facial erythema and flushing.

First Author [Ref.], year	Type of Study	n	BoNT Doses	Follow-Up	Results
Al-Niaimi F 2020 [67]	Prospective	20	In a 5 ml dilution of 500 units using typically 20–50 units per cheek or onabotulinum (BotoxTM, Allergan, Irvine, CA, USA) at 2.5 mL dilution in 100 units with doses ranging from 10 to 20 units per cheek.	3,9 months	All patients experienced improvement of erythema (documented by a 3D Antera camera)
Friedman 2019 [68]	Retrospective	16	100 U of abobotulinumtoxin after Tixel treament	1,3,6 months	flushing and erythema improvement (photographic assessment)
Park, 2015 [65]	Case report	2	3 U in chin and the eyebrow area were injected; after 1 week, 5 U in each cheek and 2 U in chin and the eyebrow area were additionally injected (patient 1) 40, 15 U in the first treatment and 5 U in the second treatment for each cheek (patient 2)	1 week to 3 months	Good improvement (photographic assessment)
Bloom, 2015 [69]	Prospective	25	15, 45 U of intradermal injections of abobotulinum toxin A into the nasal tip, nasal bridge, and nasal alae	3 months	The treatment resulted in statistically significant improvement in erythema grade at 1, 2, and 3 months after treatment when compared with baseline (3-grade scale of erythema severity on photographic assessment)
Geddoa, 2013 [64]	Pilot prospective	22	2 U per injection point with maximum dose of 100 U (neck and/or chest)	4 weeks	Twenty patients (90.9%) reported immediate improvement, and the remaining 2 patients had a second treatment session to achieve similar responses; at 4 weeks follow-up significant improvement in quality of life was measured with DLQI score
Odo, 2011 [63]	RCT	60	500 U abobotulinum toxin A, 6.2 U injection at each selected point in the skin (40 injection points of face, chest, neck, scalp); for the control group, saline solution was used at the same volume of 0.04 mL per injection point	6 months	The symptoms were less severe than before treatment; in the control group, there was no significant difference in mean intensity of sweating or in the mean number of hot flashes
Oh, 2011 [70]	RCT	15	BoNT-B doses NA; one side of the face was treated with BoNT-B, the other side with saline	8 weeks	Ineffective; mexameter demonstrated significant improvement of erythema at 8 weeks after injections on both sides; the BoNT-B injection side did not show a significant decrease in objective erythema, compared with the control side; subjective satisfaction did not differ between the treated side and the control side
Alexandroff, 2006 [61]	Case report	2	10 U spaced/hemifacial 1 cm between injections	6 weeks	No improvement was noted 6 weeks after treatment
Kranendonk 2005 [62]	Case report	1	2 U in midcheek region	Not reported	Paralysis of the zygomaticus major; no improvement after 1 week
Yuraitis, 2004 [60]	Case report	1	A total of 10 U were distributed at 1-cm increments to each cheek in the areas of the most prominent erythema	2 weeks	Marked improvement and high satisfaction

**Table 3:** Studies on the use of BoNT-A therapy in Raynaud phenomenon.

First Author [Ref.], year	Type of Study	n	BoNT-A Doses/Hand	Follow-Up	Retreatment	Results
Quintana Castanedo [84], 2020	Prospective	8	_	_	No	Reduction in pain and in the frequency of RP episodes (7 patients) any changes (1 patient)
Winter [83], 2020	Case series	4	40–300	3–21	Yes (50%)	Improved up to one year after treatment.
Dhaliwal [89], 2019	Prospective	40	100 across both hands reconstituted with 2 mL of normal saline by a single surgeon	6–12	no	Improved (Colour change and pain, swelling reduction)
Berk-Krauss [82], 2018	Case report	1	20	1, 3, 6 weeks	no	Improved pain Ulceration healed
Medina [79], 2018	Retrospective	15	100 botulinum toxin units type A in 5 mL of saline serum to 0.9% (dilution: 20 IU/mL).	1 week 1, 3, 6, 12 months	no	Improved pain Ulceration healed (70%)
Dhaliwal [81], 2018	Case reports	3	10	6 weeks	no	Improved (pain, colour changes and cold intolerance) Thermographic imaging assessed
Bello [86], 2017	RCT doubleblind; placebo	40	50 units in 2.5 mL	1, 4 months	no	Improved (pain) No changes in blod flow. Moor LDI2-IR scanner assessed)
Motegi [94], 2017	Randomized trial single-blind no placebo	45	250, 1000 or 2000 (U) of BoNT-B	16 weeks	No	Improved (pain, DU)
Motegi [90], 2016	Prospective, case series	10	10 U/finger	16 weeks	No	Improved (pain, DU, skin temperature)
Zhao [80], 2015	Case series	2	200–280	1 week, 5 months	No	Improved (pain, colour change, skin temperature)
Uppal [92], 2014	Prospective	20	100 U	6 months	No	Improved (pain with VAS, DU)
Jenkins [85], 2013	RCT pilot; doubleblind; placebo	8	40 U	–	No	Increase in digital pulp temperature
Todberg [95], 2018	Case report	1	100 U	–	No	Patient reported improvement in pain and DU
Neumeister [96], 2010 Neumeister [78], 2009	Retrospective case series	33	50 U	1–6 years	Yes (21%)	100% of DU healed, relief in 85% of patients
Fregene [87], 2009	Retrospective case series	26	20, 100 U	18 months	Yes (20%)	48% of DU healed, 35% pain reduction (VAS)
Kossintseva [93], 2008	Case report	1	100 U	12 months	No	Pain decreased, DU not reported
Van Beek [77], 2007	Retrospective case series	11	50, 200 U	9.6 months	Yes (45%)	100% decrease in pain (VAS), 82% of DU healed
Sycha [76], 2004	Pilot to RCT, case report	2	12, 300 U	–	No	37% pain reduced in 1 patient (VAS), other unknown

**Table 4:** Studies on the use of BoNT-A therapy in pompholyx.

First Author [Ref.], year	Type of Study	n	BoNT-A Doses/Hand	Follow-Up	Retreatment	Results
Ismail [102], 2020	Prospective non-randomized side-by-side comparative study	40	100	1, 4, 6 months	No	Improvement
Kontochristopoulos [101], 2007	Case reports	2	4.0 mL saline in 100 U BoNT-A; 100 U/hand	8 weeks	No	Improvement
Swartling [96], 2002	Prospective	10	1.0 mL saline in 100 U BoNT-A; mean of 162 U/hand	28–59 days	No	7 of 10 patients reported a good result; improving in VAS for itching and disease activity score
Wollina [100], 2002	Prospective; side-by-side monotherapy with topical steroid vs. adjuvant BoNT-A injection	6	2.0 mL saline in 100 U BoNT-A	8 weeks	No	Six of 6 hands treated with topical steroids in combination with BoNT-A showed improvement; BoNT-A showed a more rapid release from itching than steroids alone

**Table 5:** Cases reporting the use of BoNT in eccrine nevus.

First Author [Ref.], year	Type of Study	n	BoNT-A Doses	Follow-Up	Retreatment	Results
Sonntag [106], 2005	Case report		200 mU Dysport	36	Yes	Improvement
Honeyman [105], 2008	Case report	1	BoNT-A, dilution in 4 mL of saline, 5 U per injection; total amount not specified	1 year	Not reported	Improvement
Lera [103], 2015	Case report	1	100 U BoNT-A in 2.5 mL of saline, 2 U per injection	9 months	Yes	Improvement
Nygaard [107], 2015	Case report	1	100 U BoNT-A (dilution not specified)	1 year	Not reported	Improvement

**Table 6:** Studies on the use of BoNT-A therapy in postherpetic neuralgia.

First Author [Ref.]	Type of Study	n	BoNT-A Doses	Follow-Up	Retreatment	Results
Ding [113], 2017	Prospective	58	50 to 100	2 weeks 1, 3, 6 months	No	75% of patients improved (Variable VAS, NPS reduction)
Jain [112], 2017	Case report	2	500 units Dysport diluted with 5 mL of normal saline, making a concentration of 100 units/mL	1, 2, 4, 8, 12, 16 weeks	No	VAS for pain decreased from 9, 10 to 1
Moon [118], 2016	Case report	2	50 U BoNT-A and bupivacaine 0.1% injected under ultrasound guide in brachial plexus	5 months	No	VAS for pain decreased from 8 to 2, 3
Li [110], 2015	Case report (ophthalmic)	1	100 U of BoNT-A in the orbital region (subcutaneous)	6 months	No	VAS for pain decreased from 8–9 to 2–3
Apalla [115], 2013	Randomized, double-blind, placebo-controlled trial	29 (4 postherpetic)	20, 190 U of BoNT-A intradermally	16 weeks	No	VAS decreasing
Emad [114], 2011	interventional study		15U per 10 cm^2^ (The amount of toxin was different for every patient: not reported) intradermally	2, 4 weeks	No	VAS decreasing
Xiao [109], 2010	Randomized, double-blind, placebo-controlled trial	60	5 U/mL of BoNT-A vs. 0.5% of lidocaine vs. 0.9% of saline	3 months	No	Decrease in VAS score and improving in sleep hours superior to control group
Sotiriou [108], 2009	Case reports	3	100 U of BoNT-A in 4 mL of saline; subcutaneous in chessboard pattern	12 weeks	No	Decrease in VAS score
Liu [116], 2006	Case report	1	100 U of BoNT-A injected in a fanning pattern	9 months	No	VAS pain reduction from 10 to 1

**Table 7:** Studies on the use of BoNT-A in notalgia paresthetica.

First Author [Ref.], year	Type of Study	n	BoNT-A Doses	Retreatment	Follow-Up	Results
Maari [126], 2014	RCT vs. placebo double-blind	20	max 200 U	No	12 weeks, then placebo arm shifted to BoNT-A; total 24 weeks	No significant difference for pruritus (VAS) and hyperpigmentation
Pèrez-Pèrez [57], 2014	Retrospective, case series	5	48–56 U	No	18 months	2 worsening pruritus, little improvement in other 3 but for only 1 month
Wallengren [125], 2010	Prospective	6	18–100 U	No	18 months	5/6 patients a mean reduction of VAS by 28% at week 6; at 18 months 1 patient had a VAS of 45%, another one was still free from itch
Weinfeld [124], 2007	Case report	2	16–24 U	Yes, 18 months later with 48 U (only 1 patient)	18 months	Improvement (patient self-assessment)

**Table 8:** Cases of Hailey–Hailey disease treated with BoNT-A.

First Author [Ref.], year	n	Sites	BoNT-A Doses	Follow-Up	Results
Lapiere [131], 2000	1	Axillae	25 U, 50 U of per axilla after 6 months	4 months at time of publication	Complete remission
Kang [129], 2002	1	Groin, axillae	100 U for each inguinal fold	6 months	Improvement
Lopez-Ferrer [134], 2012	3	Axillae groin breast, axilla, axillae and groin	80 U/axilla 200 U total	5 months	All patients improved but needed at least one retreatment after 1–3 months
Charlton [135], 2017	1	Axillae and groin	50 U for axillae 50 U for groin Once a year	2 years	This therapy has restricted his disease activity to 1–2 episodes per year
Kothapalli [136], 2018	NA	Axillae and groin	50 units per axilla or groin	NA	Improvement

**Table 9:** Studies reporting the BoNT-A treatment in hidradenitis suppurativa.

First Author [Ref.], year	Type of Study	n	BoNT-A Doses	Follow-Up	Retreatment	Results
O’Reilly [150], 2005	Case report	1	250 U Dysport/axilla	10 months	No	Complete remission
Feito-Rodriguez [151], 2009	Case report	1	40 U total dose (inguinal folds)	6 months	Yes	Complete remission
Khoo [152], 2014	Case report	3, but only 1 described	50 U/axilla	3 years	Yes (3 other times)	Complete remission
Shi [153], 2019	Case report	1	100 units for each area (bilateral axillary, inframammary and groin)	NA	Yes (5 total injections)	Resolution of inflammation and healing of draining sinuses
Campanati [154], 2019	Case report	2	50 U per axilla 100 U for each side of groin	1 year	Yes (for patient 1, after 10 months after the first injection)	Real improvement
Grimstad [156], 2020	Randomised, Double-Blind, Placebo-Controlled Pilot Study	20	150 U/armpit, 200 U/groin, and 600 U in the perianal/perigenital areas	6 months	Yes (3 months after the first injection)	Clear improvement of the quality of life

**Table 10:** Androgenetic alopecia and BoNT injection therapy. FNS, finasteride.

First Author [Ref.], year	Type of Study	n	BoNT Doses	Follow-Up	Retreatment	Results
Freund [168], 2010	Open-label pilot study	50 male	150 U	60 weeks	Two treatment cycles of 24 weeks each	Treatment response rate was 75 percent
Singh [169], 2017	Open-label pilot study	10 male	150 U	6 months	No	Of 10 patients, 8 had good to excellent response on photographic assessment
Zhang [170], 2019	Open-label pilot study	24 male	50 U	6 months	No	Variable results
Shon [172], 2020	Open-label pilot study	18 male	30 U	6 months	Yes (every 4 weeks for 24 weeks).	Air density was significantly improved after 6 months but not after 3 months.
Zhou [173], 2020	Opena label, randomized study (BoNT-A vs. BoNT-A+Finasteride)	63 male and 1 woman	100 U	1 year	Yes (every 3 months)	BTA combined with FNS presents excellent results

**Table 11:** Studies on the use of BoNT-A in psoriasis. RCT, randomized clinical trial; VAS, Visual Analogue Scale; TCS Total Clinical Score.

First Author [Ref.], year	Type of Study	n (Type of Psoriasis)	BoNT-A Doses	Retreatment	Follow-Up	Results
Zanchi [178], 2008	Observational, no RCT	15 (inverse psoriasis)	50-100 U	Not reported	12 weeks	Improvement in VAS scale score; photographic assessment with improvement of erythema, infiltration (87%)
Saber [181], 2011	Case report	1 (inverse psoriasis and hyperhidrosis)	100 U per axilla	No	4 weeks	Greatly improved (photographic documentation)
Gilbert [182], 2014	Case report	1 (plaque psoriasis)	30 U for a single plaque	No	8 months	Complete remission but recurrence after 8 months.
Todberg [186], 2018	Exploratory, multicenter, randomized double-blinded trial (BoNT-A vs. vehicle).	8 (plaque psoriasis)	36 U for a single plaque	No	8 weeks	No clinical or histological differences from the vehicle
Aschenbeck [179], 2018	Open-label pilot study	8 (plaque psoriasis)	Variable number of units per plaque (average units, 53; range, 25–92)	No	10 weeks	PASI and BSA reduction
González [185], 2018	Descriptive cross-sectional study	8 (plaque psoriasis)	Variable number of units per plaque (maximum 50 U)	No	4 weeks	All parameters evaluated (desquamation, erythema and infiltration) for TCS score showed improvement
